# Estimating tuberculosis drug resistance amplification rates in high-burden settings

**DOI:** 10.1186/s12879-022-07067-1

**Published:** 2022-01-24

**Authors:** Malancha Karmakar, Romain Ragonnet, David B. Ascher, James M. Trauer, Justin T. Denholm

**Affiliations:** 1 Computational Biology and Clinical Informatics, Baker Heart and Diabetes Institute, Melbourne, VIC Australia; 2 Structural Biology and Bioinformatics, Department of Biochemistry, University of Melbourne, Melbourne, VIC Australia; 3 Victorian Tuberculosis Program and Department of Microbiology and Immunology, Doherty Institute of Infection and Immunity, University of Melbourne, 792 Elizabeth Street, Melbourne, VIC 3000 Australia; 4 School of Public Health and Preventive Medicine, Monash University, Melbourne, Australia; 5 Department of Infectious Diseases, University of Melbourne, Melbourne, VIC Australia

**Keywords:** Drug resistant tuberculosis, Epidemiological modelling, Fitness cost, Tuberculosis transmission dynamics, Resistance amplification

## Abstract

**Background:**

Antimicrobial resistance develops following the accrual of mutations in the bacterial genome, and may variably impact organism fitness and hence, transmission risk. Classical representation of tuberculosis (TB) dynamics using a single or two strain (DS/MDR-TB) model typically does not capture elements of this important aspect of TB epidemiology. To understand and estimate the likelihood of resistance spreading in high drug-resistant TB incidence settings, we used epidemiological data to develop a mathematical model of *Mycobacterium tuberculosis* (*Mtb*) transmission.

**Methods:**

A four-strain (drug-susceptible (DS), isoniazid mono-resistant (INH-R), rifampicin mono-resistant (RIF-R) and multidrug-resistant (MDR)) compartmental deterministic *Mtb* transmission model was developed to explore the progression from DS- to MDR-TB in The Philippines and Viet Nam. The models were calibrated using data from national tuberculosis prevalence (NTP) surveys and drug resistance surveys (DRS). An adaptive Metropolis algorithm was used to estimate the risks of drug resistance amplification among unsuccessfully treated individuals.

**Results:**

The estimated proportion of INH-R amplification among failing treatments was 0.84 (95% CI 0.79–0.89) for The Philippines and 0.77 (95% CI 0.71–0.84) for Viet Nam. The proportion of RIF-R amplification among failing treatments was 0.05 (95% CI 0.04–0.07) for The Philippines and 0.011 (95% CI 0.010–0.012) for Viet Nam.

**Conclusion:**

The risk of resistance amplification due to treatment failure for INH was dramatically higher than RIF. We observed RIF-R strains were more likely to be transmitted than acquired through amplification, while both mechanisms of acquisition were important contributors in the case of INH-R. These findings highlight the complexity of drug resistance dynamics in high-incidence settings, and emphasize the importance of prioritizing testing algorithms which allow for early detection of INH-R.

**Supplementary Information:**

The online version contains supplementary material available at 10.1186/s12879-022-07067-1.

## Background

Despite being both a preventable and curable disease, more than 10 million people develop tuberculosis (TB) each year, with 1.4 million deaths in 2019 [1]. Although 63 million lives have been saved through improvements in programmatic TB management this century, the increase in drug-resistant (DR-TB) cases is increasingly concerning [1]. Multidrug-resistant TB [MDR-TB; defined as resistance to both first-line drugs isoniazid (INH) and rifampicin (RIF)] is a particular barrier to TB control efforts [2]. In 2019, 465,000 people were diagnosed with MDR-TB [1]. MDR-TB can be acquired by transmission (primary resistance) or develop in vivo through inadequate or incomplete treatment (secondary resistance), and the relative contribution of these mechanisms is likely to vary by context [3]. In all settings, though, careful optimization of both clinical and public health management of MDR-TB is required to ensure good outcomes.

Mathematical modeling is increasingly used to support programmatic optimization for TB [4–6]. Accounting appropriately for DR-TB in mathematical models of disease is critical, as it differs considerably from drug-sensitive TB (DS-TB) in both epidemiological parameters and relevant outcomes. Some variation in disease characteristics is relatively well-understood, including the prolonged treatment duration [7], adverse event rates [8] and diagnostic pathway performance [9, 10]. However, considerable uncertainties persist regarding important characteristics of MDR-TB, including pathogen’s fitness, transmissibility and risk of resistance amplification related to treatment [11, 12]. Attempts to better characterize these features of MDR-TB have been challenging, in part due to the diversity of gene mutations which may confer resistance, many of which have limited clinical and epidemiological outcome data to inform model parameterization. Computational biological approaches have recently been used to bridge this gap, providing tools to estimate the fitness and resistance impact of novel TB mutations [13–15].

Modeling also offers an opportunity to quantify amplification and transmission of drug-resistant TB, by fitting dynamic models to observed data. We therefore aimed to incorporate epidemiological data into an empirically calibrated model, in order to explore parameter estimation for drug resistance amplification and transmission associated with both INH and RIF.

## Methods

### Constructing the mathematical model and defining epidemiological parameters

We designed a deterministic compartmental model of *Mtb* transmission to capture five mutually exclusive health states with regards to TB infection and disease—susceptible (S), early latent (L_A_), late latent (L_B_), infectious (I) and recovered (R, 16). The model included four TB strains: drug-susceptible (DS-TB, compartment subscript S), isoniazid mono-resistant (INH-R, compartment subscript H), rifampicin mono-resistant (RIF-R, compartment subscript R) and MDR-TB (compartment subscript M). It is to be noted that the strains are not phylogenetically related.

We assumed homogenous mixing in a closed population:

1 $$N = S + L_{{{\text{AS}}}} + L_{{{\text{AH}}}} + L_{{{\text{AR}}}} + L_{{{\text{AM}}}} + L_{{{\text{BS}}}} + L_{{{\text{BH}}}} + L_{{{\text{BR}}}} + L_{{{\text{BM}}}} + I_{{\text{S}}} + I_{{\text{H}}} + I_{{\text{R}}} + I_{{\text{M}}} + R$$

All deaths are replaced as new births (rate π) entering the susceptible compartment. This includes both deaths due to TB disease (μ_i_), as well as a universal population-wide death rate (μ).

When individuals in a population are infected with *Mtb*, they transition from the susceptible compartment (S) to the early latent compartment (L_A_). The force of infection (λ) associated with each strain (Eq. 2) is defined as:

2 $$\lambda_{X} = r_{X} \times \beta \times I_{X}$$

where “x” indexes the drug resistance pattern—S, H, R or M.

β is the “effective contact rate” for DS-TB, defined as the product of the average number of contacts between two individuals per unit time and the probability of DS-TB transmission per contact. The relative transmissibility of the different strains is denoted ${r}_{X}$ and uses the DS-TB strain’s transmissibility as reference (${r}_{S}=1$). In other words, ${r}_{X}$ represents the TB strains’ relative fitness.

People entering the early latent compartment (L_A_) can either progress (within two-years) directly to the active disease compartment (I) at rate ε, or transition to the late latent compartment (L_B_) at rate κ. Progression from L_B_ to the active disease state occurs at a much slower rate (ν), and is referred to as reactivation. Once individuals have entered the infectious compartment, one of the following six processes can occur: (1) the person may be correctly identified as having active DS-TB and commenced on treatment (rate τ), thence progressing towards cure and transitioning to the recovered (R) compartment; (2) person may be correctly identified to have DS-TB or DR-TB and commenced on treatment but experiences treatment failure without experiencing resistance amplification to other drugs and stay in the same infectious compartment; (3) spontaneous recovery (rate γ) with transition to the recovered compartment (R); (4) TB-related death (μ_i_) (5) dying of natural causes or (6) the infecting strain could acquire resistance (α_H_ and/or α_R_) to isoniazid (INH-R), rifampicin (RIF-R) or MDR-TB and move to I_H_, I_R_ and ultimately to I_M_ compartments. To capture the progressive accrual of resistance with each transition, only one level of additional resistance not already present can be obtained during a disease episode. People who have spontaneously recovered from past TB or successfully completed treatment are both represented as a single compartment (R) on the assumption that prognosis is equivalent regardless of the infecting strain from which each person recovers. Once treatment is complete, the recovered person can transition back to L_A_ through reinfection, represented as δ. We define δ (Eq. 3) as:

3 $$\delta_{X} = RR_{r} * \lambda_{X}$$

where, RR_r_ is the “relative risk of re-infection once recovered”.

Latently infected people also have a risk of re-infection with the same or other strains represented as θ in the model; and the re-infecting strain would “override” the existing strain. We define θ_x_ (Eq. 4) similarly to δ_x_ as:

4 $$\theta_{X} = RR_{i} * \lambda_{X}$$

where, RR_i_ is the “relative risk of re-infection once latently infected”.

**Fig. 1:**
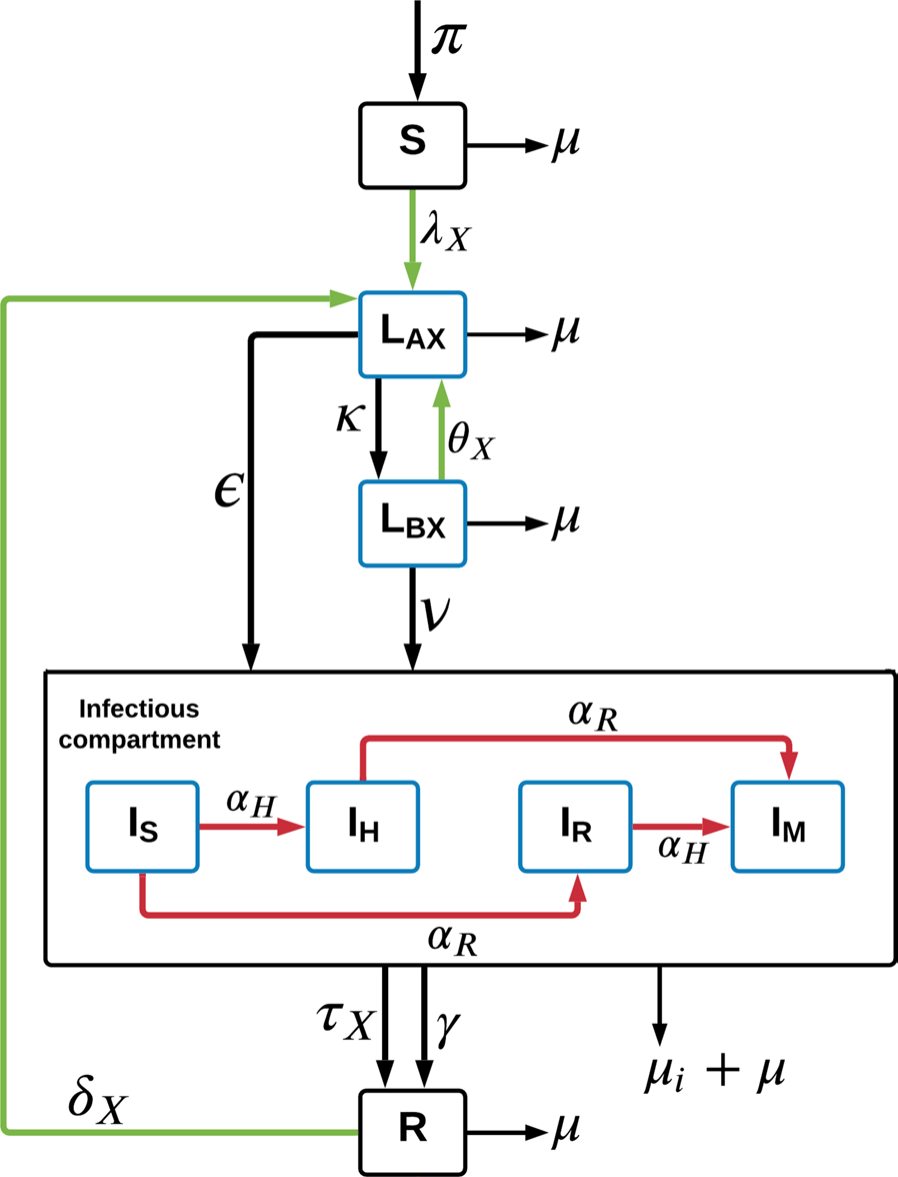
Structure of four strain Mtb transmission model. The symbols S, L_A_, L_B_, I and R represent uninfected/susceptible, early latent, late latent, infected and recovered health states, respectively. The subscript “X” used in L_A_ and L_B_ compartments and other parameters, indexes the drug resistance patterns, with S, H, R and M representing susceptible, isoniazid mono-resistance, rifampicin mono-resistance and multidrug resistance respectively. The infectious compartment is elaborated in the figure to show the amplification flows of INH and RIF respectively, parameterized with α_H_ and α_R_ (red arrows). The green arrows represent infection/transmission flows, black arrows represent constant progression flows. Compartments stratified according to resistance profiles are shown in blue. (π–birth rate, λ_X_—force of infection, ε–rate of early progression, κ–rate of late progression, ν–reactivation rate, γ–spontaneous recovery rate, θ_X_–risk of re-infection once latently infected, μ–mortality rate, μ_i_–TB-specific mortality rate, τ_X_–treatment rate, δ_X_—risk of re-infection after recovery)

Figure 1 and Additional file 1: figure S1 which is a representation of the model, along with Eqs. 5–18, captures all the intermediary steps and parameters as explained in the paragraph above.

It is to be noted that the figure does not show individuals who are latently infected with a given strain will have the same strain if they develop active disease. An elaborated diagram in presented in the supplementary sheets where all the compartments modelled have been shown (Additional file 1: Fig. S1).

Ordinary differential equations used to define the four-strain model

5 $$\frac{dS}{{dt}}{ } = \pi { } - { }\left( {\lambda_{S} + \lambda_{H} + \lambda_{R} + \lambda_{M} + \mu } \right) S$$

6 $$\frac{{dL_{AS} }}{dt} = \lambda_{S} S - \left( { \in { } + { }\kappa { } + { }\mu } \right)L_{AS} + \theta_{S} \left( {L_{BS} + L_{BH} + L_{BR} + L_{BM} } \right) + \delta_{S} R$$

7 $$\frac{{dL_{AH} }}{dt} = { }\lambda_{H} S{ } - { }\left( { \in { } + { }\kappa { } + { }\mu } \right)L_{AH} + \theta_{H} \left( {L_{BS} + L_{BH} + L_{BR} + L_{BM} } \right) + \delta_{H} R$$

8 $$\frac{{dL_{AR} }}{dt} = \lambda_{R} S{ } - { }\left( { \in { } + { }\kappa { } + { }\mu } \right)L_{AR} + \theta_{R} \left( {L_{BS} + L_{BH} + L_{BR} + L_{BM} } \right) + \delta_{R} R$$

9 $$\frac{{dL_{AM} }}{dt} = \lambda_{M} S{ } - { }\left( { \in { } + { }\kappa { } + { }\mu } \right)L_{AM} + \theta_{M} \left( {L_{BS} + L_{BH} + L_{BR} + L_{BM} } \right) + \delta_{M} R$$

10 $$\frac{{dL_{BS} }}{dt} = \kappa L_{AS } - \left( {\nu + \theta_{S} + \theta_{H} + \theta_{R} + \theta_{M} + \mu } \right)L_{BS}$$

11 $$\frac{{dL_{BH} }}{dt} = \kappa L_{{AH{ }}} - { }\left( {\nu + \theta_{S} + \theta_{H} + \theta_{R} + \theta_{M} + \mu } \right)L_{BH}$$

12 $$\frac{{dL_{BR} }}{dt} = \kappa L_{{AR{ }}} - { }\left( {\nu + \theta_{S} + \theta_{H} + \theta_{R} + \theta_{M} + \mu } \right)L_{BR}$$

13 $$\frac{{dL_{BM} }}{dt} = { }\kappa L_{{AM{ }}} - { }\left( {\nu + \theta_{S} + \theta_{H} + \theta_{R} + \theta_{M} + \mu } \right)L_{BM}$$

14 $$\frac{{dI_{S} }}{dt} = \in L_{{AS{ }}} + { }\nu L_{BS} - \alpha_{H} I_{S} - \alpha_{R} I_{S} { } - \left( {\gamma + \tau_{S} + \mu_{i} + \mu } \right)I_{S}$$

15 $$\frac{{dI_{H} }}{dt} = \in L_{{AH{ }}} + { }\nu L_{BH} + \alpha_{H} I_{S} { } - \alpha_{R} I_{H} { } - \left( {\gamma + \tau_{H} + \mu_{i} + \mu { }} \right)I_{H}$$

16 $$\frac{{dI_{R} }}{dt} = { } \in L_{{AR{ }}} + { }\nu L_{BR} - \alpha_{H} I_{R} { } + \alpha_{R} I_{S} - \left( {\gamma + \tau_{R} + \mu_{i} + \mu } \right)I_{R}$$

17 $$\frac{{dI_{M} }}{dt} = \in L_{AM } + \nu L_{BM} + \alpha_{H} I_{R} + \alpha_{R} I_{H} - \left( {\gamma + \tau_{M} + \mu_{i} + \mu } \right)I_{M}$$

18 $$\frac{dR}{{dt{ }}} = \left( {\tau_{S} + \gamma } \right)I_{S} + \left( {\tau_{H} + \gamma } \right)I_{H} + \left( {\tau_{R} + \gamma } \right)I_{R} + \left( {\tau_{M} + \gamma } \right)I_{M} - { }\left( {\delta_{S} + { }\delta_{H } + \delta_{R} + \delta_{M} + { }\mu } \right)R$$

### Parameter estimation

Parameters can be categorized as universal, country-specific and time-variant parameters, as presented in Table 1.

**Table 1:** Epidemiological parameters used for calibrating the model and their prior distribution ranges

A) Universal parameters			
Parameter	Value	Prior distribution	Sources
Early progression (ε) (year^−1^)	0.401775	Uniform [0.1–0.8]	[17]
Transition to late latency (κ) (year^−1^)	3.6525	Uniform [1.0–7.0]	[17]
Reactivation (ν) (year^−1^)	0.002008875	Uniform [0.0009, 0.006]	[17]
Spontaneous recovery (γ) (year^−1^)	0.2	Gamma [0.16, 0.29], mode = 0.20	[18]
Natural mortality (μ) (year^−1^)	0.0142		
TB-specific mortality (μ_i_) (year^−1^)	0.2	Gamma [0.06, 1.06], mode = 0.08	[18]
Relative risk of reinfection once infected	0.21	–	[19]

B) Country-specific and time-variant parameters (used for model calibration)				
Parameter	Country		Prior distribution	Sources
	The Philippines	Viet Nam		
Transmission rate (β)	[1–35]	[1–30]	Uniform	Fitted
Fitness cost of INH-R TB strain	[0.50–1.20]		Uniform	[20], [21]
Fitness cost of RIF-R TB strain	[0.50–1.20]		Uniform	[22], [12]
Fitness cost of MDR-TB strain	[0.50–0.99]		Uniform	[23]
Proportion of failures developing RIF-R TB (ρ_R_)	[0.01–0.99]		Uniform	Fitted
Proportion of failures developing INH-R TB (ρ_H_)	[0.01–0.99]		Uniform	Fitted
Relative risk of reinfection once recovered	[0.50–1.50]		Uniform	Fitted
CDR start time	[1950 -1970]		Uniform	Fitted
CDR final value	[0.30–0.80]		Uniform	Fitted

*CDR* case detection rate

#### Universal parameters

From the literature we gathered information on disease-specific and epidemiological parameters to calibrate the *Mtb* transmission model. We considered these parameters to be universal to all TB settings and so assigned the same values for all strains and settings (Table 1A).

Once we defined the parameters in our model, we next reviewed literature for information on the prior distributions of uncertain parameters (Table 1B).

#### Defining time-variant model processes

To capture the rise of drug resistance over time, we allowed the case detection rate (CDR, a proportion, defined in Eq. 20) and the treatment success rate (TSR) to vary with time. TSR is the probability of a person being first tested and ultimately put on treatment to be cured, or simply put the probability of treatment success at presentation. This parameter was further varied by strain.

People diagnosed with active TB are commenced on treatment upon identification and move from the infectious compartments (I, I_H_, I_R_ and I_M_) to the recovered compartment (R). The transition from the infectious to the recovered compartment is represented using the parameter “τ”. τ is dependent on the TB detection rate “*d*” and the TSR and is mathematically expressed in Eq. 19.

19 $${\tau }\left( t \right) = d\left( t \right)*{\text{TSR }}\left( t \right)$$

where, “d” is calculated by solving the following CDR equation:

20 $$CDR\left( t \right) = \frac{d\left( t \right)}{{d\left( t \right) + {\upgamma } + { }\mu_{i} + \mu }}$$

which can be re-written as:

21 $$d\left( t \right) = \frac{CDR\left( t \right)}{{\left( {1 - CDR\left( t \right)} \right)*\left( {\gamma + \mu_{i} + \mu } \right)}}$$

(* “t” represents time in Eqs. 19, 20 and 21).

Sigmoidal functions were used to model progressive increases for both the CDR and the TSR between 1950 and 2020. The final value of the TSR was set to the most recent TSR estimate reported by the WHO. In contrast, the final value of the CDR was varied during calibration. This allowed flexibility in simulating the historical dynamics of TB control in the countries considered.

#### Defining the amplification rate

Treatment for tuberculosis begins once individuals are detected with TB and the TB strain is correctly identified. Treatment then proceeds and may result in three possible outcomes: death, successful treatment or treatment failure. Treatment failure can further be associated with acquiring new resistance to one additional drug that was not previously present in the infecting organism. INH and RIF are part of the standard regimen for the treatment of drug-susceptible strains. Gain in resistance to either INH or RIF is represented using amplification rates α_H_ or α_R_ respectively in the model. Mathematical representation of INH and RIF mono-resistant amplification is shown in Eqs. 22 and 23.

22 $${\text{Rate}}\,{\text{of}}\,{\text{amplification }}\left( {\alpha_{H} } \right)\, = { }d \left( t \right)*\left( {1{ } - {\text{ TSR }}\left( t \right)} \right){*}\rho_{H}$$

23 $${\text{Rate of amplification }}\left( {\alpha_{R} } \right) = { }d \left( t \right)*\left( {1{ } - {\text{ TSR }}\left( t \right)} \right){*}\rho_{R}$$

where, *ρ*_*H*_ = Proportion of previously INH-susceptible individuals that acquire resistance on treatment failure, *ρ*_*R*_ = Proportion of previously RIF-susceptible individuals that acquire resistance on treatment failure, and “t” stands for time.

### Model calibration to prevalence and notification data

#### Prevalence data

The model presented above was calibrated to country-specific data. We fitted the models using TB prevalence estimates from national TB prevalence (NTP) surveys (Viet Nam: 2006–2007 and 2017–2018; The Philippines: 2007 and 2016) and drug-resistance prevalence from national DR-TB surveys (DRS, Viet Nam: 2011; The Philippines: 2009, 2016). The detailed estimates are presented in Table 2.

**Table 2:** Summaries of prevalence survey results and drug resistance survey data for Philippines and Viet Nam

A) TB prevalence data				
Country	Year	TB prevalence (per 100, 000)	95% CI	Sources
Viet Nam	2006–2007	307.2	248.8–365.6	[24]
	2017–2018	322	260–399	[25]
The Philippines	2007	660	510–810	[26]
	2016	1159	1016–1301	[27]

B) Drug resistance data					
Country	Drug resistance	Year	Drug resistance (%)	95% CI	Sources
Viet Nam	Isoniazid mono resistance	2011	14.86	12.15–17.56	[28]
	Rifampicin mono resistance	2011	0.23	0.1–0.35	[28]
	MDR-TB	2011	6.93	4.22–9.63	[28]
The Philippines	Isoniazid mono resistance	2009	9.44	7.95–10.92	[29]
	Rifampicin mono resistance	2009	1.008	0.71–1.304	[29]
	MDR-TB	2009	5.8	4.3–7.5	[29]
The Philippines	Isoniazid mono resistance	2016	12.43	11.1–13.75	[27]
	Rifampicin mono resistance	2016	0.82	0.44–1.19	[27]
	MDR-TB	2016	3.35	2.53–4.41	[27]

#### Notification data

We used WHO-reported TB notifications as a calibration target for both models. For Viet Nam, in 2018, 102,171 cases were notified and for The Philippines 382,543 cases were notified and we calibrated to the per capita notification rates corresponding to these values.

### Uncertainty analysis

An Adaptive Metropolis (AM) algorithm was used to estimate model parameters [30], including drug resistance amplification rates. An AM algorithm adapts continuously to the target distribution. It uses the history of the process to tune the effective proposal distribution suitably. The size and the spatial distribution of the proposal distribution is significantly affected due to adaptation. Moreover, AM is easy to implement and use, with no additional computational cost. As soon as the simulation process starts, AM algorithms start using the cumulating information. This gives the algorithm a major advantage because at an early stage of the simulation, the rapid start of adaptation enables the search to be more effective. This even diminishes the number of function evaluations needed [30].

The AM algorithm [30] was used to generate samples from the posterior distribution of the parameters from 25,000 iterations for each country. The primary estimates are reported as the posterior median value for all parameters of interest such as amplification proportions, CDR, relative fitness of each modelled strain and the relative risk of infection once recovered (δ). The intervals reported are obtained by calculating the 25th and 75th percentile of each parameter’s posterior distribution. Programming was done in Python 3.7.3 and all code and associated data are publicly available on GitHub (github.com/malanchak/AuTuMN).

## Results

### Calibration of the model

**Fig. 2:**
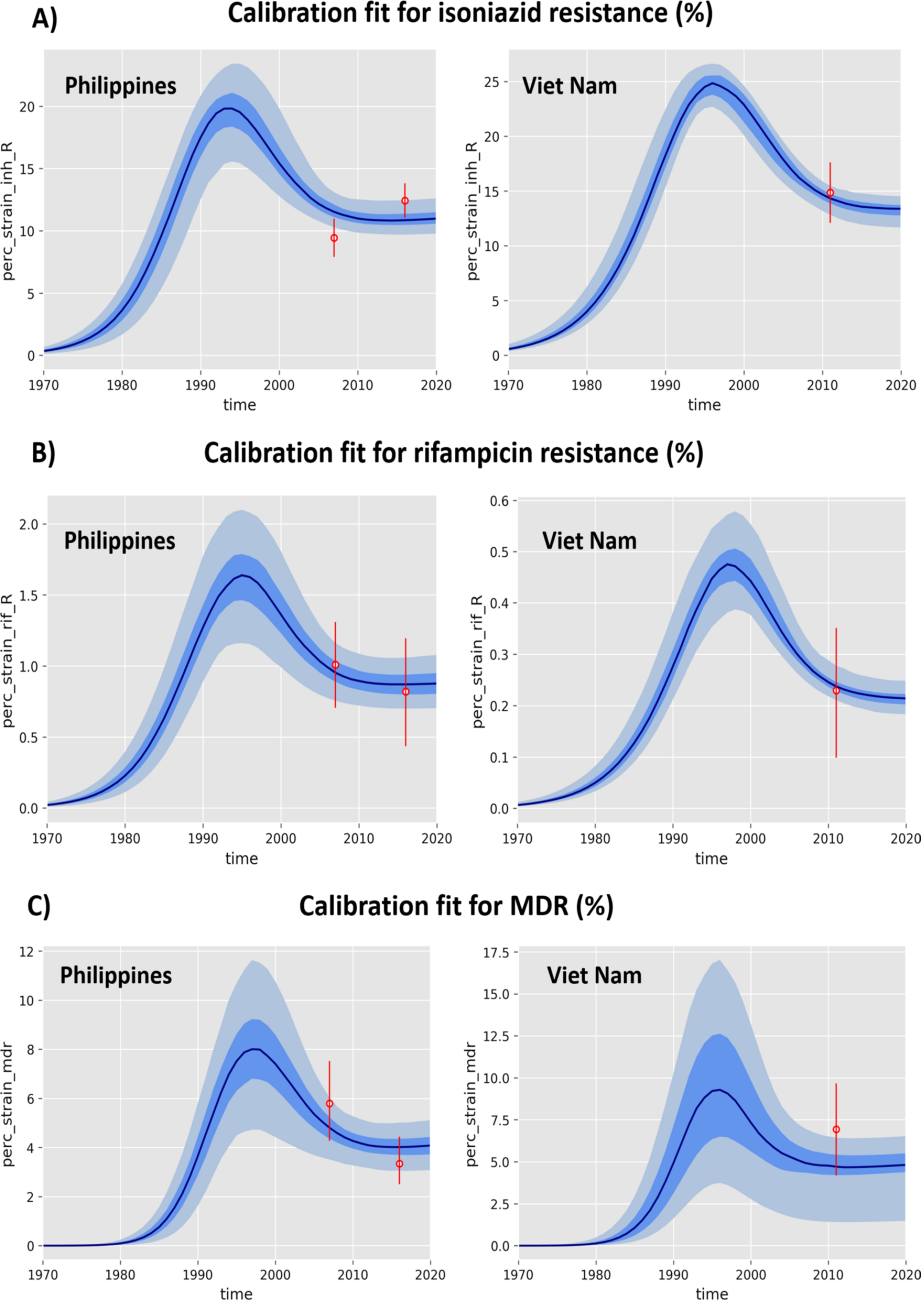
Model calibration: **A** Isoniazid mono resistance, **B** Rifampicin mono resistance and **C** MDR-TB. The red dots with the line represent the empiric data (including intervals) obtained from the drug resistance surveys of the Philippines and Viet Nam. The model predictions are represented in blue solid line as median, interquartile range (dark blue shade) and central 95% credible interval (light blue shade)

Figure 2A–C show the model fits to reported INH-R, RIF-R and MDR levels for the high DR-TB settings, The Philippines and Viet Nam respectively.

Table 3 shows the posterior distributions of all calibrated parameters.

**Table 3:** Posterior distribution of parameters obtained using the AM algorithm

DR- TB related parameter	Estimate (median, 50% CI)	
	The Philippines	Viet Nam
Proportion of previously INH-susceptible individuals that acquire resistance on treatment failure	0.84 (0.79–0.89)	0.77 (0.71–0.84)
Proportion of previously RIF-susceptible individuals that acquire resistance on treatment failure	0.05 (0.04–0.07)	0.011 (0.010–0.012)
Relative fitness of INH-R TB strains	0.87 (0.83–0.92)	0.98 (0.95–1.00)
Relative fitness of RIF-R TB strains	0.78 (0.74–0.84)	0.77 (0.73–0.81)
Relative fitness of MDR-TB strains	0.67 (0.58–0.71)	0.64 (0.56–0.75)
CDR final/maximum value	0.49 (0.47–0.51)	0.66 (0.63–0.69)

Universal parameters	Estimate (median, 50% CI)	
	The Philippines	Viet Nam
Rate of rapid progression (ε) (year-1)	0.33 (0.28–0.37)	0.22 (0.19–0.29)
Rate of transition towards late latency (κ) (year-1)	5.49 (4.78–5.95)	3.62 (3.13–4.92)
Rate of re-activation (ν) (year-1)	0.003 (0.002–0.004)	0.0017 (0.0016–0.0018)
Relative risk of re-infection after recovery (δ)	0.74 (0.64–0.86)	0.64 (0.56–0.81)

### Drug resistance amplification and transmission

**Fig. 3:**
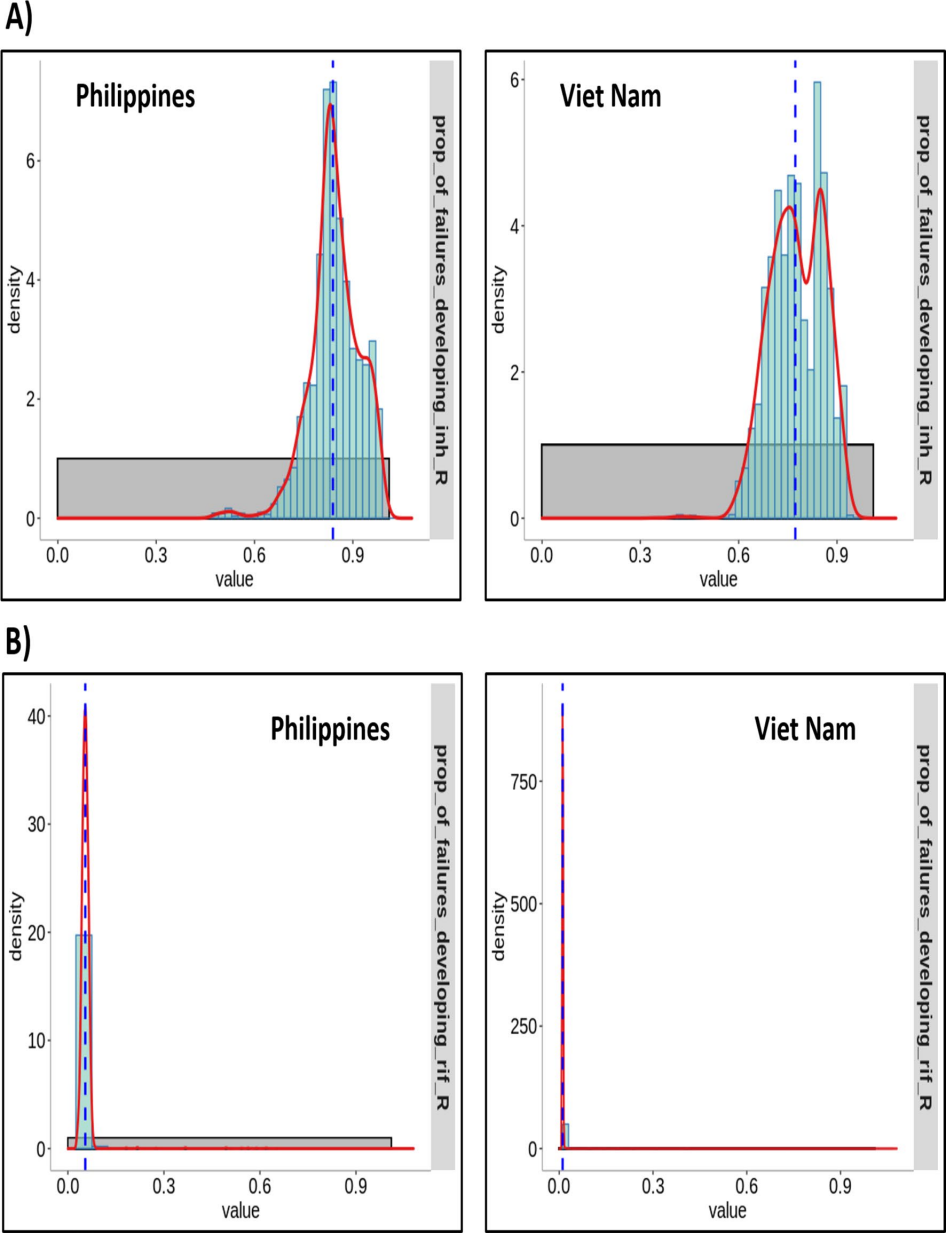
The estimated risk of INH-R and RIF-R amplification when treatment fails. The probability density function (red line) represents the posterior distribution of the estimates of amplification and the white background represents the prior ranges. The dashed blue line is the median of the estimates. **A** Proportion of previously INH-susceptible strains that acquire resistance on treatment failure and **B** proportion of previously RIF-susceptible strains that acquire resistance on treatment failure

We observed higher proportions of drug resistance amplification for INH compared to RIF for both the high DR-TB incidence settings we simulated (Fig. 3). The estimated risk of INH-R amplification when treatment fails was 0.84 (95% CI 0.79–0.89) for The Philippines and 0.77 (95% CI 0.71–0.84) for Viet Nam. The estimated risk of RIF-R amplification when treatment fails was 0.05 (95% CI 0.04–0.07) for The Philippines and 0.011 (95% CI 0.010–0.012) for Viet Nam. This meant approximately 84% and 77% of the people who failed treatment in The Philippines and Viet Nam respectively would end up with resistance to INH.

The model was used to estimate the proportions of incident DR-TB due to transmission compared to DR amplification (Table 4). In the Philippines, the proportions of incident INH-R TB due to transmission was 50% (43–70), RIF-R TB was 52% (43–70) and MDR-TB was 40% (28–52). For Viet Nam, the proportions of incident INH-R TB due to transmission was 67% (54–73), RIF-R TB was 63% (55–71) and MDR-TB was 43% (34–51).

**Table 4:** Estimates obtained for proportions of incident DR-TB due to direct transmission rather than DR amplification

DR-TB	Estimate (median, 50% CI)	
	The Philippines	Viet Nam
INH-R TB	50 (43–70)	67 (54–73)
RIF-R TB	52 (43–70)	63 (55–71)
MDR-TB	40 (28–52)	43 (34–51)

In The Philippines, the model estimates for amplification from DS to INH-R was 26 per 100,000 (95% CI 15–33) people, followed by 10 per 100,000 (95% CI 6–14) people then gain resistance to RIF and moving to the MDR compartment. Comparing this to acquiring RIF resistance first, we see 2 per 100,000 (95% CI 1–3) moving from DS to RIF-R, followed by only 0.05 (95% CI 0.02–0.08) people gaining resistance to INH to move to the MDR compartment. A similar observation was seen for Viet Nam, the model estimates for amplification from DS to INH-R shows 6 per 100,000 (95% CI 4–8) people followed by people 4 per 100,000 (95% CI 3–5) gaining resistance to RIF and moving to the MDR compartment. In case of DS to RIF-R transition the estimates were 0.08 per 100,000 (0.06–0.11) people, followed by 0.0007 per 100,000 (0.0004–0.001) people gaining resistance to INH to move to the MDR compartment.

### Estimates for relative strain fitness and CDR

The posterior estimates of relative fitness associated with INH-R strains for The Philippines was 0.87 (95% CI 0.83–0.92) and 0.98 (95% CI 0.95–1.00) for Viet Nam. The relative fitness associated with RIF-R strains for The Philippines was 0.78 (95% CI 0.74–0.84) and 0.77 (95% CI 0.73–0.81) for Viet Nam. The relative fitness associated with MDR-TB strains in The Philippines was 0.67 (95% CI 0.58–0.71) compared to 0.64 (95% CI 0.56–0.75) for Viet Nam.

Our study also provided information on estimates of CDR with high precision for both the settings, as inclusion of notification and prevalence of infection data for the analysis helped in constraining the parameter. The estimates obtained for The Philippines was 0.49 (95% CI 0.47–0.51) and for Viet Nam was 0.66 (95% CI 0.63–0.69).

## Discussion

From this modeling study we were able to construct a model which successfully replicated epidemiological dynamics in two high burden TB settings, incorporating parameters drawn from microbiological fitness data. Using this model to explore the development of drug resistance in these contexts, we found that a much higher proportion of treatment failure resulted in amplification for INH-R rather than for RIF-R. This finding is consistent with observed higher rates of INH-R globally [31, 32] and allows consideration of factors which might be mechanistically important for understanding and planning a programmatic response. For example, in a prevalence survey from 1975, the rate of INH-R in Canadians with TB following an initial course of therapy was 75.6% [33].

One factor likely to play a significant role in preferential INH-R amplification is current methods of DR-TB detection which prioritize RIF’s resistance identification. According to WHO and many country guidelines, TB patients with strains found to be resistant to RIF need to start on a recommended MDR-TB treatment regimen. Longer MDR-TB regimens, and historical second-line therapy regimens, frequently have INH included in them, irrespective of resistance to INH being either undetermined or confirmed. Re-treatment regimens in particular have often incorporated prolonged durations of INH therapy—for example the category II regimen used in the Philippines comprised of 8 months of INH, RIF and ethambutol supplemented by streptomycin for the initial 2 months, and pyrazinamide for the initial 3 months (2SHRZE/HRZE/ 5HRE) [34], and older treatment regimens used in Viet Nam comprised of 8 months of INH and ethambutol supplemented by initial two months of streptomycin, pyrazinamide and rifampicin (2SHRZ/6HE, 28). These factors may be further amplified by use of isoniazid in the private sector and/or through community pharmacy settings, where worse guideline adherence and increased risk resistance development has been shown [35, 36] but with poorer treatment outcomes compared to NTPs [37, 38].

In addition to programmatic insights, our model provides novel information on parametrizing CDR. This is important, as this parameter cannot be measured directly yet plays a significant role in informing robust mathematical model of TB transmission. As with any mathematical representation our model has certain limitations. Our model was calibrated to TB prevalence and DR surveys to estimate the risk of resistance amplification. But the definition of a TB case may change between surveys, even within the same country. We have adopted a simplified model structure that does not capture factors such as age, comorbidities and other heterogeneity associated with TB epidemics. These factors may affect the risk of resistance amplification. Our model is primarily built for pulmonary tuberculosis and does not include extra-pulmonary TB data, as our primary focus was on transmission. For the same reason, this model has been parametrized from adult TB data given the limited TB transmission from young children to others. In our model we assumed the risk of INH-R amplification is the same starting from I_S_, as compared to starting from I_R_; the same applies for RIF-R amplification. We even assumed the fitness cost of MDR-TB is independent of that of INH-R of RIF-R. Therefore, these limitations can potentially influence the estimated risk of resistance amplification.

Furthermore, it is difficult to compare the results generated by our model with existing studies because the findings are novel and not many researchers have tried to estimate risk of monoresistance amplification of INH using a four strain TB transmission model. Two studies which reported similar findings were—(1) study conducted in the KwaZulu-Natal Province of South Africa for extremely drug resistant TB transmission, reported 84% of the participants failed treatment [39]. (2) A study from Kampala, Uganda found proportion of patients that acquired resistance were low, to which the author mentions that the study only reflects a single cycle of treatment in a heavily treated cohort of patients and therefore their results may underestimate the degree of drug-resistance amplification [40]. With respect to estimates for transmission, the results are comparable to the study conducted by Kendall et al. where they report estimated percentage of MDR-TB resulting from transmission varied substantially with different countries notification data; for example, 48% (30–75%) in Bangladesh versus 99% (91–100%) in Uzbekistan [41].

Historically, diagnosis of MDR-TB has been reliant on culture-based phenotypic testing, which in high-burden settings may be applied selectively, such as after treatment failure. As part of the global policy to control DR-TB, many high burden settings have pledged to deploy the molecular diagnostic assay Xpert MTB/RIF (detects resistance only in RIF), which is a nucleic acid amplification test that can be directly applied to sputum samples [42, 43]. As the presence of RIF resistance is highly predictive of MDR-TB, these policies have led to significant improvements in the appropriate initiation of second-line therapy [44]. However, as our work highlights, these algorithms may also be associated with selecting for and further amplifying INH resistance. Alternative molecular tools, such as the line probe assay MTBDR*plus* [45], Abbott RealTime MTB RIF/INH (Abbott), FluoroType MTBDR [46], BD MAX MDR-TB assay [47], Roche cobas MTB [48] and whole genome sequencing can identify both RIF and INH resistance, and may offer the programmatic advantages of rapid MDR-TB diagnosis while avoiding this secondary effect [49]. Further research into the association between specific INH resistance mutations and differential risk of transmission will be helpful in better defining the public health impact of this effect [50].

An interesting observation was made between the estimates of the fitness cost of each resistant strain and their respective transmission dynamics for both the countries. We see that the RIF-R and MDR-TB have similar fitness in both countries indicating homogeneity of the population, while INH-R strains seem to have a considerable difference between the two countries. This is reflected in the transmission dynamics observed for the INH-R strains, where Viet Nam has a higher transmission rate compared to The Philippines. This could mean the INH-R strains in Viet Nam, although under significant drug pressure in the host, are more transmissible, perhaps due to strain-specific factors or local host/pathogen interaction. The same correlation cannot be made for RIF-R strains. They have similar fitness cost in both countries, but the transmission rate is higher in Viet Nam compared to The Philippines.

Comparing fitness cost of INH-R strains to the proportions of individuals who develop INH-R due to treatment failure (ρ_H_) for both the high burden DR-TB setting, we see that the fitness cost is inversely related to ρ_H_. While INH-R strains in Viet Nam have a higher fitness cost than The Philippines, the number of individuals acquiring resistance due to treatment failure is lower in Viet Nam. The estimates for CDR were also higher in Viet Nam compared to The Philippines. As, ρ_H_ and CDR is directly proportional to the amplification rate as defined in Eq. 22, we can say that the amplification rate α_H_ is inversely proportional to the fitness cost of INH-R strains. Therefore, an increase in the mycobacterial fitness for INH-R can lead to a potential increase in transmission rate but a decrease in the amplification rate. In case of RIF-R strains, the fitness cost was similar for both the settings. The slight difference in the amplification rate can be explained by the higher CDR observed in Viet Nam. Factors such as age, behavior, and gender ratio of the overall population, could possibly be a reason to see this type of differences. But it is beyond the scope of this paper to predict the effects of these external influences on this model.

## Conclusion

While rapid molecular diagnostics will continue to be important for programmatic adoption, it is also important to recognize that the principle of unrecognized resistance amplification demonstrated here can be repeated for any resistance not routinely addressed in diagnostic algorithms. It is therefore essential to incorporate genome sequencing into surveillance programs, to maximize the clinical and public health benefits [51]. With recent developments in next generation sequencing techniques, we have now have high-throughput diagnostic tools for the detection of DR-TB which are both fast and efficient [52]. While such tools are currently in routine use only in high resource settings, the benefits associated with these tools should be prioritized for high burden contexts to support optimal individual and program outcomes [53, 54].

## Supplementary Information

**Additional file 1: Figure S1.** A detailed representation of the Mtb transmission model 

## Data Availability

All data generated or analysed during this study are included in this published article. The code is available at the github repository—github.com/malanchak/AuTuMN.

## References

[1] WHO. Global Tuberculosis Report 2020. https://www.whoint/teams/global-tuberculosis-programme/tb-reports/global-tuberculosis-report-2020. 2020.

[2] WHO. WHO consolidated guidelines on drug-resistant tuberculosis treatment. 2019. 30946559

[3] Ragonnet R, Trauer JM, Denholm JT, Marais BJ, McBryde ES (2017). High rates of multidrug-resistant and rifampicin-resistant tuberculosis among re-treatment cases: where do they come from?. BMC Infect Dis.

[4] Trauer JM, Ragonnet R, Doan TN, McBryde ES (2017). Modular programming for tuberculosis control, the “AuTuMN” platform. BMC Infect Dis.

[5] Zwerling A, Shrestha S, Dowdy DW (2015). Mathematical Modelling and Tuberculosis: Advances in Diagnostics and Novel Therapies. Adv Med.

[6] Fors J, Strydom N, Fox WS, Keizer RJ, Savic RM (2020). Mathematical model and tool to explore shorter multi-drug therapy options for active pulmonary tuberculosis. PLoS Comput Biol.

[7] Pontali E, Raviglione MC, Migliori GB (2019). Regimens to treat multidrug-resistant tuberculosis: past, present and future perspectives. Eur Respir Rev.

[8] Herrera M, Bosch P, Nájera M, Aguilera X (2013). Modeling the spread of tuberculosis in semiclosed communities. Comput Math Methods Med.

[9] Wikell A, Åberg H, Shedrawy J, Röhl I, Jonsson J, Berggren I (2019). Diagnostic pathways and delay among tuberculosis patients in Stockholm, Sweden: a retrospective observational study. BMC Public Health.

[10] Naidoo P, van Niekerk M, du Toit E, Beyers N, Leon N (2015). Pathways to multidrug-resistant tuberculosis diagnosis and treatment initiation: a qualitative comparison of patients' experiences in the era of rapid molecular diagnostic tests. BMC Health Serv Res.

[11] Becerra MC, Huang C-C, Lecca L, Bayona J, Contreras C, Calderon R (2019). Transmissibility and potential for disease progression of drug resistant Mycobacterium tuberculosis: prospective cohort study. BMJ.

[12] Knight GM, Colijn C, Shrestha S, Fofana M, Cobelens F, White RG (2015). The distribution of fitness costs of resistance-conferring mutations is a key determinant for the future burden of drug-resistant tuberculosis: a model-based analysis. Clin Infect Dis.

[13] Karmakar M, Globan M, Fyfe JAM, Stinear TP, Johnson PDR, Holmes NE (2018). Analysis of a novel pncA mutation for susceptibility to pyrazinamide therapy. Am J Respir Crit Care Med.

[14] Karmakar M, Rodrigues CHM, Horan K, Denholm JT, Ascher DB (2020). Structure guided prediction of Pyrazinamide resistance mutations in pncA. Sci Rep.

[15] Karmakar M, Rodrigues CHM, Holt KE, Dunstan SJ, Denholm J, Ascher DB (2019). Empirical ways to identify novel Bedaquiline resistance mutations in AtpE. PLoS ONE.

[16] Trauer JM, Denholm JT, McBryde ES (2014). Construction of a mathematical model for tuberculosis transmission in highly endemic regions of the Asia-pacific. J Theor Biol.

[17] Ragonnet R, Trauer JM, Scott N, Meehan MT, Denholm JT, McBryde ES (2017). Optimally capturing latency dynamics in models of tuberculosis transmission. Epidemics.

[18] Ragonnet R, Flegg JA, Brilleman SL, Tiemersma EW, Melsew YA, McBryde ES, et al. Revisiting the natural history of pulmonary tuberculosis: a Bayesian estimation of natural recovery and mortality rates. Clin Infect Dis 2020. 10.1093/cid/ciaa602 32766718

[19] Trauer JM, Moyo N, Tay EL, Dale K, Ragonnet R, McBryde ES (2016). Risk of active tuberculosis in the five years following infection … 15%?. Chest.

[20] Cohen T, Sommers B, Murray M (2003). The effect of drug resistance on the fitness of *Mycobacterium tuberculosis*. Lancet Infect Dis.

[21] Borrell S, Gagneux S (2009). Infectiousness, reproductive fitness and evolution of drug-resistant *Mycobacterium tuberculosis*. Int J Tubercul Lung Dis.

[22] Gagneux S (2009). Fitness cost of drug resistance in *Mycobacterium tuberculosis*. Clin Microbiol Infect.

[23] Cohen T, Murray M (2004). Modeling epidemics of multidrug-resistant *M. tuberculosis* of heterogeneous fitness. Nature Med.

[24] Hoa NB, Sy DN, Nhung NV, Tiemersma EW, Borgdorff MW, Cobelens FG (2010). National survey of tuberculosis prevalence in Viet Nam. Bull World Health Organ.

[25] Nguyen HV, Tiemersma EW, Nguyen HB, Cobelens FGJ, Finlay A, Glaziou P (2020). The second national tuberculosis prevalence survey in Vietnam. PLoS ONE.

[26] Tupasi TE, Radhakrishna S, Chua JA, Mangubat NV, Guilatco R, Galipot M (2009). Significant decline in the tuberculosis burden in the Philippines ten years after initiating DOTS. Int J tuberc Lung Dis.

[27] Department of Health RoTP. National Tuberculosis Prevalence Survey 2016 Philippines. 2016; http://www.ntp.doh.gov.ph/downloads/publications/Philippines_2016%20National%20TB%20Prevalence%20Survey_March2018.pdf.

[28] Nhung NV, Hoa NB, Sy DN, Hennig CM, Dean AS (2015). The fourth national anti-tuberculosis drug resistance survey in Viet Nam. Int J Tuberc Lung Dis.

[29] Nationwide drug resistance survey of tuberculosis in the Philippines. Int J Tuberc Lung Dis 2009;13(4):500–7. 19335957

[30] Haario H, Saksman E, Tamminen J (2001). An adaptive metropolis algorithm. Bernoulli.

[31] Jenkins HE, Zignol M, Cohen T (2011). Quantifying the burden and trends of isoniazid resistant tuberculosis, 1994–2009. PLoS ONE.

[32] Mahmoudi A, Iseman MD (1993). Pitfalls in the care of patients with tuberculosis: common errors and their association with the acquisition of drug resistance. JAMA.

[33] Eidus L, Jessamine AG, Hershfield ES, Helbecque DM (1978). A national study to determine the prevalence of drug resistance in newly discovered previously untreated tuberculosis patients as well as in retreatment cases. Can J Public Health = Revue canadienne de sante publique..

[34] Chiang C-Y, Trébucq A (2018). Tuberculosis re-treatment after exclusion of rifampicin resistance. Eur Respir J.

[35] Quy HT, Lan NT, Lönnroth K, Buu TN, Dieu TT, Hai LT (2003). Public-private mix for improved TB control in Ho Chi Minh City, Vietnam: an assessment of its impact on case detection. Int J Tuberc Lung Dis.

[36] Tupasi TE, Radhakrishna S, Co VM, Villa ML, Quelapio MI, Mangubat NV (2000). Bacillary disease and health seeking behavior among Filipinos with symptoms of tuberculosis: implications for control. Int J Tuberc Lung Dis.

[37] Lönnroth K, Thuong LM, Lambregts K, Quy HT, Diwan VK (2003). Private tuberculosis care provision associated with poor treatment outcome: comparative study of a semi-private lung clinic and the NTP in two urban districts in Ho Chi Minh City, Vietnam. National Tuberculosis Programme. Int J Tuberc Lung Dis.

[38] Buu TN, Lönnroth K, Quy HT (2003). Initial defaulting in the National Tuberculosis Programme in Ho Chi Minh City, Vietnam: a survey of extent, reasons and alternative actions taken following default. Int J Tuberc Lung Dis.

[39] Shah NS, Auld SC, Brust JCM, Mathema B, Ismail N, Moodley P (2017). Transmission of extensively drug-resistant tuberculosis in South Africa. N Engl J Med.

[40] Temple B, Ayakaka I, Ogwang S, Nabanjja H, Kayes S, Nakubulwa S (2008). Rate and amplification of drug resistance among previously-treated patients with tuberculosis in Kampala, Uganda. Clin Infect Dis.

[41] Kendall EA, Fofana MO, Dowdy DW (2015). Burden of transmitted multidrug resistance in epidemics of tuberculosis: a transmission modelling analysis. Lancet Respir Med.

[42] Tsara V, Serasli E, Christaki P (2009). Problems in diagnosis and treatment of tuberculosis infection. Hippokratia.

[43] Campbell EA, Korzheva N, Mustaev A, Murakami K, Nair S, Goldfarb A (2001). Structural mechanism for rifampicin inhibition of bacterial rna polymerase. Cell.

[44] Dlamini MT, Lessells R, Iketleng T, de Oliveira T (2019). Whole genome sequencing for drug-resistant tuberculosis management in South Africa: what gaps would this address and what are the challenges to implementation?. J Clin Tuberc Other Mycobact Dis..

[45] Nathavitharana RR, Hillemann D, Schumacher SG, Schlueter B, Ismail N, Omar SV (2016). Multicenter noninferiority evaluation of hain GenoType MTBDRplus Version 2 and Nipro NTM+MDRTB line probe assays for detection of rifampin and isoniazid resistance. J Clin Microbiol.

[46] de Vos M, Derendinger B, Dolby T, Simpson J, van Helden PD, Rice JE (2018). Diagnostic accuracy and utility of FluoroType MTBDR, a new molecular assay for multidrug-resistant tuberculosis. J Clin Microbiol.

[47] Hofmann-Thiel S, Plesnik S, Mihalic M, Heiß-Neumann M, Avsar K, Beutler M (2020). Clinical evaluation of BD MAX MDR-TB assay for direct detection of *Mycobacterium tuberculosis* complex and resistance markers. J Mol Diagn.

[48] Nadarajan D, Hillemann D, Kamara R, Foray L, Conteh OS, Merker M (2021). Evaluation of the Roche cobas MTB and MTB-RIF/INH assays in samples from Germany and Sierra Leone. J Clin Microbiol.

[49] Talbot EA, Pai M (2019). Tackling drug-resistant tuberculosis: we need a critical synergy of product and process innovations. Int J Tuberc Lung Dis.

[50] Fregonese F, Ahuja SD, Akkerman OW, Arakaki-Sanchez D, Ayakaka I, Baghaei P (2018). Comparison of different treatments for isoniazid-resistant tuberculosis: an individual patient data meta-analysis. Lancet Respir Med.

[51] Dunstan SJ, Williamson DA, Denholm JT (2019). Understanding the global tuberculosis epidemic: moving towards routine whole-genome sequencing. Int J Tuberc Lung Dis.

[52] Mahomed S, Naidoo K, Dookie N, Padayatchi N (2017). Whole genome sequencing for the management of drug-resistant TB in low income high TB burden settings: challenges and implications. Tuberculosis (Edinb).

[53] Luo T, Yang C, Peng Y, Lu L, Sun G, Wu J (2014). Whole-genome sequencing to detect recent transmission of Mycobacterium tuberculosis in settings with a high burden of tuberculosis. Tuberculosis (Edinb).

[54] Sulis G, Pai M (2020). Isoniazid-resistant tuberculosis: a problem we can no longer ignore. PLoS Med.

