# Supplementary material for: Estimating tuberculosis drug resistance amplification rates in high-burden settings
Source: BMC Infect Dis. 2022 Jan 24;22:82. doi: 10.1186/s12879-022-07067-1 (PMC8785585; doi:10.1186/s12879-022-07067-1)
Supplement: Supplementary file 1 — Additional file 1: Figure S1. A detailed representation of the Mtb transmission model [file 12879_2022_7067_MOESM1_ESM.docx]

Supplementary Data

Estimating the risk of tuberculosis drug resistance amplification in high-burden settings

# Malancha Karmakar^1,2,3^, Romain Ragonnet^4^, David B. Ascher^1,2^, James M. Trauer^4^, Justin T. Denholm^3,5 *^

# ^1^ Computational Biology and Clinical Informatics, Baker Heart and Diabetes Institute, Melbourne, Victoria, Australia

# ^2^ Structural Biology and Bioinformatics, Department of Biochemistry, University of Melbourne, Melbourne, Victoria, Australia

# ^3^ Victorian Tuberculosis Program and Department of Microbiology and Immunology, Doherty Institute of Infection and Immunity, University of Melbourne, Melbourne, Victoria, Australia

# ^4^ School of Public Health and Preventive Medicine, Monash University, Melbourne, Australia

# ^5^ Department of Infectious Diseases, University of Melbourne, Melbourne, Victoria, Australia

# * To whom correspondence should be addressed J.T.D 792 Elizabeth Street, Melbourne Victoria Australia 3000. Tel: +61 3 9342 9428; Email: [justin.denholm@mh.org.au](mailto:justin.denholm@mh.org.au)

S1: A detailed representation of the Mtb transmission model


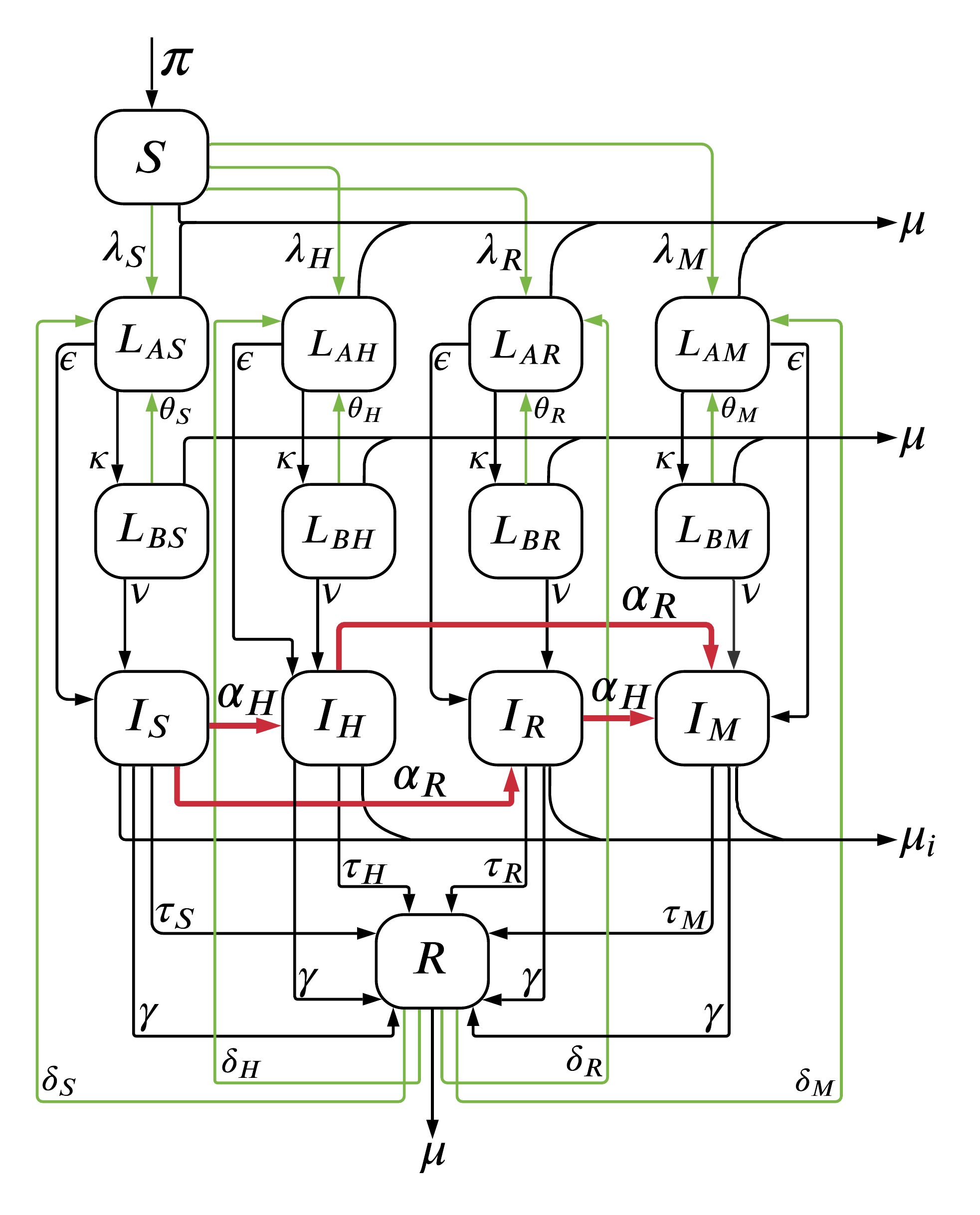


The detailed model structure of the four strain TB transmission model. We developed a compartmental deterministic model of TB transmission which captures the five mutually exclusive health states with regards to TB infection and disease. These are susceptible (S), early latent (L_A_), late latent (L_B_), infectious (I) and recovered (R). The four strains included in the model are - drug-susceptible (compartment subscript S), isoniazid mono-resistant (compartment subscript H), rifampicin mono-resistant (compartment subscript R) and multidrug resistance TB (compartment subscript M). The amplification flows of INH and RIF, α_H_ and α_R_ respectively, are shown in red arrows. The green arrows represent infection/transmission flows, black arrows represent constant progression flows.

The various parameters included in the model are as follows:

π – birth rate, λ - force of infection, ε – rate of early progression, κ – rate of late progression, ν – reactivation rate, γ – spontaneous recovery rate, θ – risk of re-infection once latently infected, μ – mortality rate, μ_i_ – TB-specific mortality rate, τ – treatment rate, δ - risk of re-infection after recovery.
